# Supplementary material for: Deletion of wfs1 Impairs Oligodendrocyte Precursor Cells Dorsal Distribution and Myelination Through the wfs1-hmgcs1 Axis in Zebrafish
Source: Biology (Basel). 2026 Aug 16;15(16):1402. doi: 10.3390/biology15161402 (PMC13510151; doi:10.3390/biology15161402)
Supplement: Supplementary file 1 [file biology-15-01402-s001.zip › File S1-Transcriptome sequencing data and differentially expressed genes (DEGs) in wfs1-- zebrafish at 5 dpf/2.KEGG/wfs1KOvsWT/UP/src/dre04270.html]

dre04270


- dre:337226

- Up regulated genes

337226\_1(4.74603512488049)
- dre:798632

- Up regulated genes

798632(1.33935841885554)

- dre:321070

- Up regulated genes

101882911(3.50047007681359)

- dre:100006675

- Up regulated genes

100006675(1.63301233543285)

- dre:436718

- Up regulated genes

436718(1.34229427553561)

- dre:492486

- Up regulated genes

492486(4.56703139609518)

- dre:192322

- Up regulated genes

336121(1.42904963424916)

- dre:393783

- Up regulated genes

393783\_1(2.31540559534787)

- dre:337226

- Up regulated genes

337226\_1(4.74603512488049)
- dre:798632

- Up regulated genes

798632(1.33935841885554)

- dre:406370

- Up regulated genes

406370(7.33922178722892)

- dre:568726

- Up regulated genes

568726(1.14419230813088)
- dre:560807

- Up regulated genes

560807(1.71247301071773)

- dre:436966

- Up regulated genes

436966\_2(1.93836605617093)
- dre:567131

- Up regulated genes

567131(1.07865220568899)

- dre:403114

- Up regulated genes

403114\_1(7.0947381564129)

Close
